# Configurable switching behavior in polymer-based resistive memories by adopting unique electrode/electrolyte arrangement†

**DOI:** 10.1039/d1ra03561d

**Published:** 2021-07-02

**Authors:** Karthik Krishnan, Shaikh Mohammad Tauquir, Saranyan Vijayaraghavan, Ramesh Mohan

**Affiliations:** Corrosion and Material Protection Division, CSIR-Central Electrochemical Research Institute (CECRI) Karaikudi TN 630-003 India karthikk@cecri.res.in; Microsystem Packaging Group, CSIR-Central Electronics Engineering Research Institute Pilani Rajasthan-333031 India

## Abstract

The difference in resistive switching characteristics by modifying the device configuration provides a unique operating principle, which is essential for both fundamental studies and the development of future memory devices. Here, we demonstrate the poly(methyl methacrylate) (PMMA)-based resistive switching characteristics using four different combinations of electrode/electrolyte arrangement in the device geometry. From the current–voltage (*I–V*) measurements, all the PMMA-based devices revealed nonvolatile memory behavior with a higher ON/OFF resistance ratio (∼10^5^–10^7^). Significantly, the current conduction in the filament and resistive switching behavior depend majorly on the presence of Al electrode and electrochemically active silver (Ag) element in the PMMA matrix. A trap-controlled space charge limited conduction (SCLC) mechanism constitutes the resistive switching in the Al/PMMA/Al device, whereas the current conduction governed by ohmic behavior influences the resistive switching in the Ag-including devices. The depth-profiling X-ray photoelectron spectroscopy (XPS) study evidences the conducting filament formation processes in the PMMA-based devices. These results with different conduction mechanisms provide further insights into the understanding of the resistive switching behavior in the polymer-based devices by simply rearranging the device configuration.

## Abstract 2

Poly(methyl methacrylate) (PMMA)-based MIM devices have been investigated using four different combinations of electrode/electrolyte arrangement in the device geometry in order to elucidate the resistive switching characteristics.

## Graphical abstract

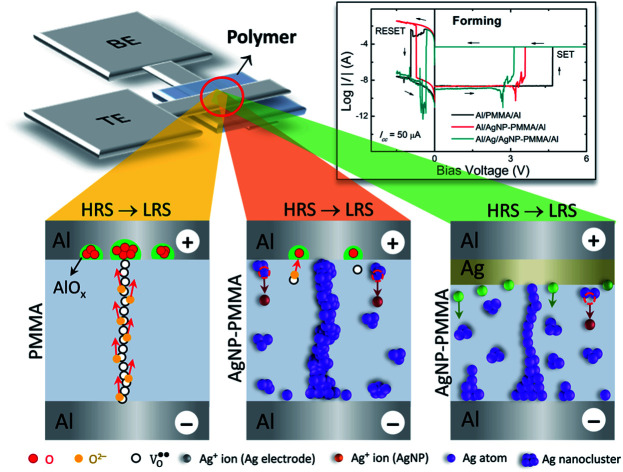

## Introduction

Easy tunable resistive switching characteristics in a metal–insulator–metal (MIM) structured device are key for the development of not only nonvolatile memories but also for future atomic electronics.^1–5^ In a MIM structured device, both organic (such as PEO, PVA, P3HT, *etc.*) and inorganic (such as Ta_2_O_5_, SiO_2_, ZnO, HfO_2_, *etc.*) materials serve as an ion conducting insulating dielectrics for resistive switching operation.^6–13^ However, the resistive switching operation mainly depends on the configuration of the electrode and electrolyte.^14–17^ It has been widely reported that the resistive switching in inorganic-based MIM devices arose by the valence change mechanism (VCM), in which the reduced oxygen atoms create oxygen vacancy sites in the dielectric interface for electron hopping between the electrodes during forward biasing, resulting in an ON state. Application of reverse biasing dissolves the conducting bridge between the electrodes due to the recombination of oxygen ions and oxygen vacancies, enabling an OFF state.^18–21^ Conversely, the organic polymer-based MIM devices operate mostly by the electrochemical metallization mechanism (ECM).^22,23^ In ECM cells, the subsequent growth and annihilation of conducting metallic bridge by the electrochemically active elements (such as Ag or Cu) in the dielectric polymer interface can be responsible for the ON and OFF state during respective biasing conditions.^24–26^ Incorporating electrochemically active elements either in electrode or electrolyte can mostly induce the filament growth and bistable resistive switching behavior. Though, the filament growth occurs randomly as stochastic events that determines the resistive switching characteristics such as forming voltage, SET and RESET voltages, ON and OFF resistance values.^27,28^ Significantly, the atomic constriction of thin filament results quantum transportation, where the lateral dimension of the conduction pathways are analogous to the Fermi wavelength.^29,30^ The highly optimized device configuration and experimental measuring conditions are prime factors influencing the atomic point contact in the conducting filament, in which the discrete quantized states (*G*_0_ = 2*e*^2^/*h*, where *e* is the charge of electron and *h* is Planck's constant) can be realized in MIM devices.^31–34^ Therefore, selecting an appropriate electrode and electrolyte material in the MIM device configuration plays crucial role in determining the filament formation and resistive switching property.

**Fig. 1:**
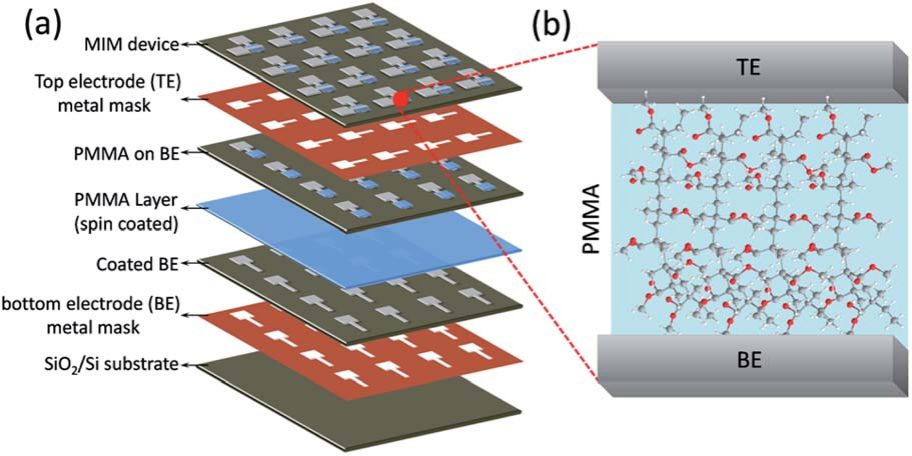
Schematics of the (a) fabrication steps for PMMA-based stacked device, and (b) cross-sectional view of the MIM device; excess polymer film area was wiped out before TE coating.

Here, the PMMA-based MIM devices have been investigated for resistive switching operation using various device configurations. Four different combinations of electrode/electrolyte arrangement in the MIM structure (Al/PMMA/Al, Al/Ag/PMMA/Al, Al/AgNP-PMMA/Al and Al/Ag/AgNP-PMMA/Al) were used to demonstrate the current conduction mechanism in the filament and resistive switching characteristics. A step by step MIM device fabrication is schematically shown in Fig. 1. Owing to its good structural ordering, better thermal stability, higher ionic conductivity and easy film forming ability, PMMA can be served as an essential solid polymer electrolyte for nonvolatile memories and other applications.^35–38^ In general, the resistive switching behavior is significantly affected by the inherent properties of the polymer matrix such as its crystalline order, ionic conductivity and thermal stability.^4,6,8^ In the PMMA-based devices, the nonvolatile memory characteristics is found with large ON/OFF resistance ratio (10^5^–10^7^). Furthermore, the combinations of electrode/electrolyte arrangement in the MIM device structure greatly alters the resistive switching characteristics, interpreted by different conduction mechanism in the filament.

## Results and discussion

### Resistive switching characteristics of PMMA-based devices

**Fig. 2:**
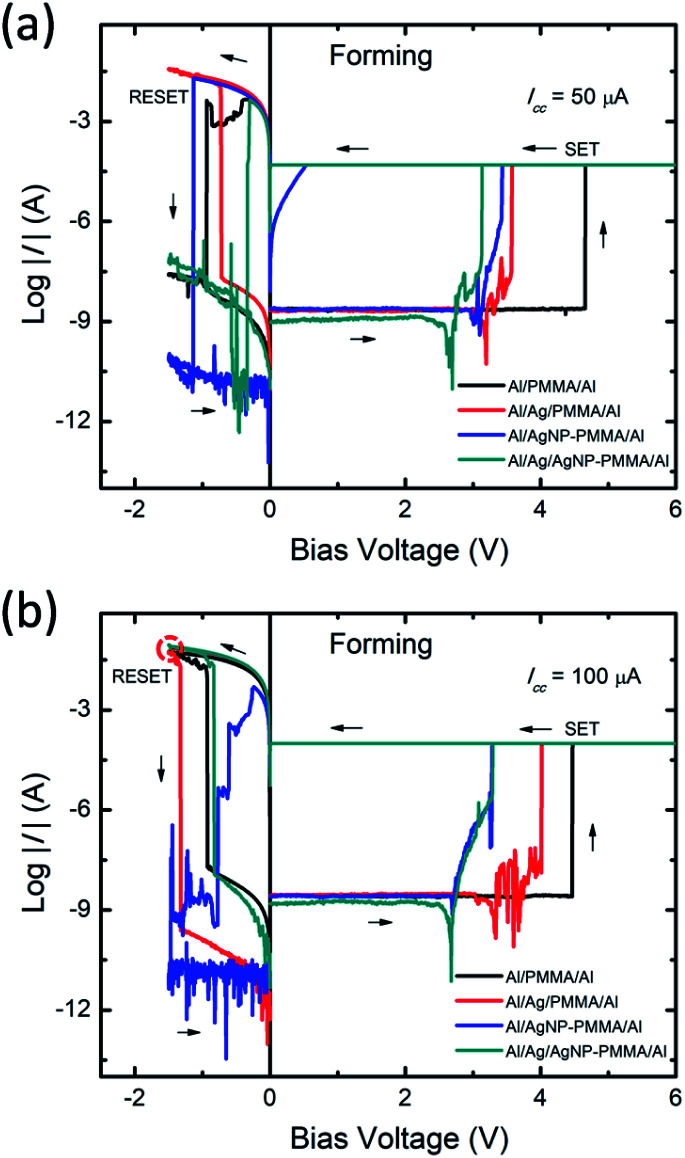
Typical *I–V* plots of the forming processes of the PMMA-based devices, measured under different *I*_CC_ ranges (a) 50 μA, and (b) 100 μA.

The current–voltage (*I–V*) behavior of PMMA-based devices have been investigated with various device configurations. Fig. 2 represents typical *I–V* plots of four different device configurations, obtained from the first sweep cycle. In all the devices, application of positive bias enables low resistance state (LRS) from high resistance state (HRS) and the process is called as SET. It is well known that, the first SET state corresponds to the initial growth of conducting metal filament between the electrodes, referred to as forming process.^39,40^ During negative bias, the dissolution of conducting bridge between the electrodes switch the device from LRS to HRS, called as RESET process. In general, the resistive switching characteristics such as SET/RESET voltages, ON/OFF resistance ratio and endurance cycles are greatly influenced by both the device configuration and *I–V* measurement parameters.^41^ Especially, the applied potential and compliance current (*I*_CC_) range plays crucial role in determining the stable switching operation.^41^

To elucidate the impact of *I*_CC_ on resistive switching behavior, the *I*_CC_ dependent forming processes were initially performed on all the devices. The *I*_CC_ was varied between 10 μA and 100 μA. In all the devices, smaller *I*_CC_ range (≤30 μA) exhibited unstable or volatile switching behavior (Fig. S1a and b, ESI†) due to the higher ON resistance (more than 10 kΩ). It is observed that the increase in *I*_CC_ influences the ON state resistance, interpreting the stability of the conducting filament.^42^ The increase in *I*_CC_ revealed stable nonvolatile switching behavior with a decrease of ON resistance value. From the *I–V* plots (Fig. 2a and b), the ON resistance values are found to be decreased from ∼200 Ω to ∼99 Ω when the *I*_CC_ varied from 50 μA to 100 μA, respectively. It suggests that, the strong filament formation between the electrodes at higher *I*_CC_ range,^42^ enabling large current flow prior to the RESET process (indicated by red dotted circle in Fig. 2b). Therefore, the optimum *I*_CC_ range required to enable stable resistive switching behavior. Our previous findings also suggested that the *I*_CC_ range below 10 μA always led to volatile switching due to thinner conductive filament and above this range led to nonvolatile switching due to thicker conductive filament between the electrodes.^41^ Moreover, the forming voltage of the devices are evaluated from the *I–V* data of five different cells at each *I*_CC_ range (Fig. S1c, ESI†). The forming voltage of Al/Ag/AgNP-PMMA/Al device shows smaller value as compared to the remaining device configurations.

**Fig. 3:**
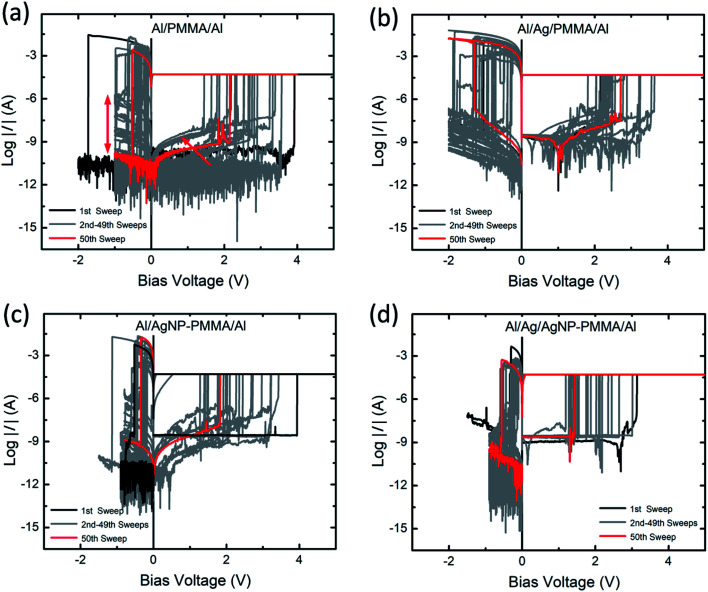
Typical *I–V* characteristics of (a) Al/PMMA/Al, (b) Al/Ag/PMMA/Al, (c) Al/AgNP-PMMA/Al, and (d) Al/Ag/AgNP-PMMA/Al stacked device, measured at a sweep rate of 5 mV s^−1^ and *I*_CC_ of 50 μA for 50 consecutive sweep cycles.

In order to understand the repeatable switching behavior of PMMA-based devices, the *I–V* measurement was carried out for more than 50 continuous sweep cycles, as seen in Fig. 3a–d. In all the cases, the nonvolatile resistive switching is observed though the SET and RESET voltages varied somewhat between the first (black curve) and 50th (red curve) sweep cycles. In the Al/PMMA/Al device (Fig. 3a), the large fluctuations in the SET (1.4 V to 3.9 V) and RESET (−0.15 V to −1.7 V) voltages are found. Especially, the RESET behavior with less prominent HRS (indicated by red double arrow) is observed during reverse biasing. It can be due to the partial rupture of the conducting channels between the electrodes.^43^ Such partial rupture of the conducting channels affects the next sweep cycle and varies the *V*_SET_ (indicated by red arrow). However, these fluctuated switching characteristics recovered certainly after few sweep cycles. In the Al/Ag/PMMA/Al device, the long cycle *I–V* plots show repeatable resistive switching behavior with *V*_SET_ ranges from 1.8 V to 3.6 V and RESET voltage (*V*_RESET_) ranges from −0.28 V to −1.8 V (Fig. 3b). A significant feature of current minimum in the *I–V* plot is observed during positive biasing (Fig. S2 of ESI†). The current minimum in a logarithmic scale analogous to a zero-current crossing point in a linear scale is associated with electric polarization. The bias voltage at this zero-current crossing point is related with an open-circuit voltage (electromotive force, emf) created by the electrochemical reactions at the electrode–polymer interfaces and distribution of charge carriers in the polymer matrix.^44,45^ As consequence, electric polarization originates from the accumulation of positive and negative charges at the opposite electrode interfaces. Similar behavior has been found in our previous studies using Ag metal-based polymer resistive switching devices.^8,25^

Fig. 3c represents the *I–V* curves of AgNP included PMMA-based device (Al/AgNP-PMMA/Al). It is found that the *V*_SET_ is distributed from 1.2 V to 3.9 V and *V*_RESET_ is varied between −0.16 V and −1.1 V. It is obvious that the inclusion of both Ag electrode and AgNP in the PMMA device (Al/Ag/AgNP-PMMA/Al) exhibited relatively smaller *V*_SET_ (1.1 V to 3.2 V) and *V*_RESET_ (−0.09 V to −0.6 V), as seen in Fig. 3d. It can be due to the large Ag^+^ ionic concentration distributed in the PMMA interface, which favors the SET and RESET operation at smaller biasing voltages for the entire sweep cycles of the Ag/Ag/AgNP-PMMA/Al device.^25^ These results indicate that the resistive switching characteristics in the PMMA-based devices is altered significantly by the device configuration.

**Fig. 4:**
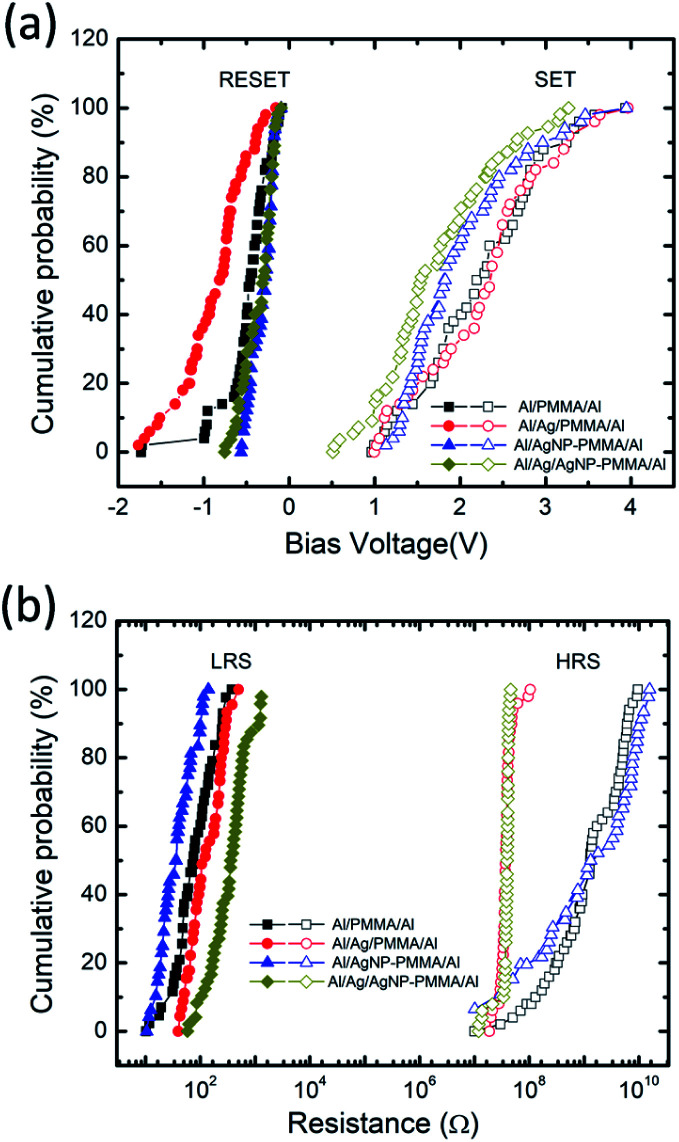
Cumulative probability plots of (a) *V*_SET_/*V*_RESET_ behavior, and (b) corresponding *R*_LRS_/*R*_HRS_ of all the four devices, measured using cycle-to-cycle variation.

The endurance characteristics of the PMMA-based devices have been analyzed by taking into account of the continuous sweep cycles. Usually, the device configuration and experimental parameters such as stop voltage, step voltage and *I*_CC_ greatly alters the SET/RESET voltages and ON/OFF resistance ratio.^25,41^ A statistical distribution of the switching voltages and resistance values are investigated during cycle-to-cycle operation. Fig. 4a and b represents the cumulative probability distributions of *V*_SET_/*V*_RESET_ and resistance states (*R*_LRS_/*R*_HRS_) for all the devices. The *V*_SET_/*V*_RESET_ were directly extracted from the continuous sweep cycle data (Fig. 3), and the corresponding the resistance values were obtained at ±0.1 V. Table S1 (ESI†) summarizes the mean value (*μ*) and standard deviation (*σ*) of the *V*_SET_/*V*_RESET_ and *R*_LRS_/*R*_HRS_ for all the four devices. It is found that the Al/Ag/AgNP-PMMA/Al device exhibits relatively small value of *μ* and *σ* in the *V*_SET_/*V*_RESET_ as compared to the remaining device configurations. By using the mean values of *R*_LRS_ and *R*_HRS_, the ON/OFF ratio is found to be ≈10^7^ for Al/PMMA/Al and Al/AgNP-PMMA/Al devices and ≈10^5^ for Al/Ag/PMMA/Al and Al/Ag/AgNP-PMMA/Al devices, respectively.

### Filament growth processes in PMMA-based devices

**Fig. 5:**
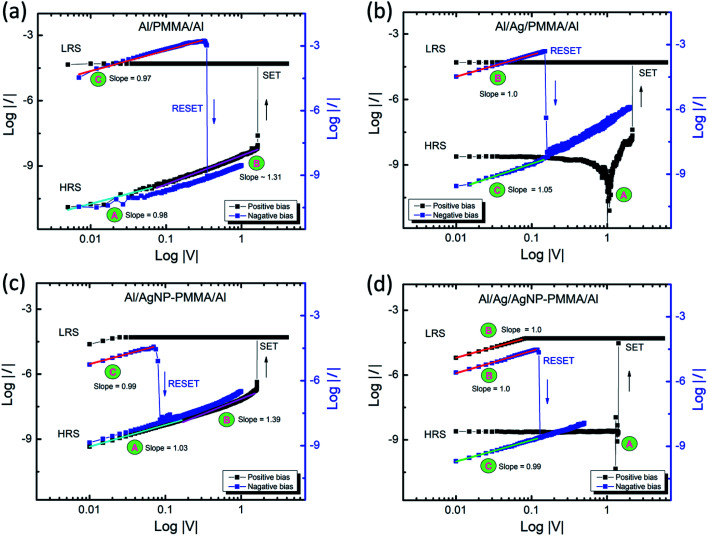
The experimental and fitted *I–V* curves of (a) Al/PMMA/Al, (b) Al/Ag/PMMA/Al, (c) Al/AgNP-PMMA/Al, and (d) Al/Ag/AgNP-PMMA/Al devices in the double logarithmic scale under both SET (positive bias) and RESET (negative bias) processes.

From the *I–V* measurements, the nonvolatile memory behavior is observed in all the four PMMA-based devices. Nevertheless, each device showed different switching characteristics such as forming voltage, *V*_SET_/*V*_RESET_ and *R*_LRS_/*R*_HRS_, as shown in Fig. 2–4. It is essential to mention that, the Al/PMMA/Al device showed resistive switching characteristics without an active metal element (Ag) in the device configuration has attracted and stimulated further onto investigate the conduction mechanism of the PMMA-based devices. The *I–V* curves of both forward and reverse bias conditions were fitted using a double logarithmic plot (log *I–*log *V*) in order to explain the current conduction mechanism in the PMMA-based devices. Fig. 5 illustrates the experimental non-linear *I–V* plot containing different regions fitted with separate linear slopes, suggesting the variation in conduction phenomena under biasing conditions. It is obvious that the conduction mechanism in PMMA-based devices without Ag electrode is entirely different from the Ag electrode included devices. According to the fitted lines, region A and region B of Al/PMMA/Al and Al/AgNP-PMMA/Al devices (Fig. 5a and c), the ohmic behavior (*I* ∝ *V*) with the Mott–Gurney law behavior (*I* ∝ *V*^2^) is observed in the HRS, inferring the charge carrier transport obeys the space charge limited conduction (SCLC) mechanism.^46,47^ Subsequent to the SET process, ohmic behavior dominates the carrier transport in the LRS (the slope ≈ 1 in the log *I–*log *V* of region C), which is a mostly observed characteristics in the conductive metal filament based memory devices.^46,48^ It is expected that, the Al top electrode oxidized to form partial AlO_*x*_ layer due to the absorbance of oxygen from the oxygen anions under positive bias, which plays major role in the switching behavior.^49,50^ The formed AlO_*x*_ in the electrode may act as the charge trap sites, which can build up a space charge by the accumulation of charge carriers at the electrode–PMMA interface.^46,51^ Similar Al electrode based resistive switching characteristics governed by SCLC conduction mechanism has already been reported.^12,52^ Furthermore, the current conduction in the Al/AgNP-PMMA/Al device closely associated with the SCLC conduction mechanism. This can be due to the presence of Al as top electrode in the device architecture, which may induce the charge trap controlled conduction mechanism. However, the presence of Ag element in the polymer matrix in general exhibits the Ag conducting filament based resistive switching.^25^

Interestingly, the Ag electrode included PMMA-devices exhibit different conducting mechanism from the other two devices, as shown in Fig. 5b and d. Almost similar *I–V* behavior is observed for both Al/Ag/PMMA/Al and Al/Ag/AgNP-PMMA/Al devices. In the log *I–*log *V* plots, three major regions are noticed. The region A in the plot corresponds to the electric polarization effect, which is a most commonly observed behavior in the polymer based ECM cells.^8,25^ The regions B and C (slope ≈ 1.0) denotes the carrier transportation strays from the ohmic conduction mechanism in the LRS and HRS, respectively.

**Fig. 6:**
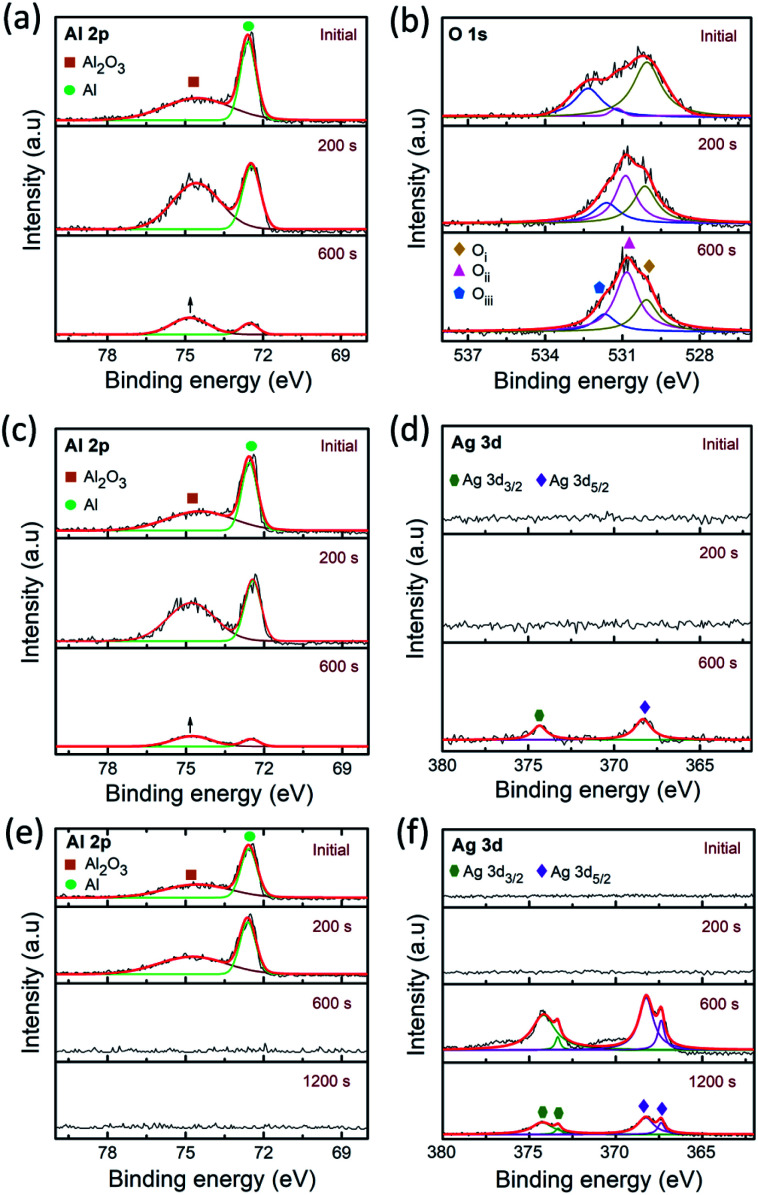
Depth-profiling XPS spectra of Al 2p, O 1s and Ag 3d signals of (a) and (b) Al/PMMA/Al (c) and (d) Al/AgNP-PMMA/Al, and (e) and (f) Al/Ag/PMMA/Al devices after biasing in LRS, respectively.

To get further insight into the filament growth processes and resistive switching behavior in the PMMA-based devices, the depth-profiling XPS analysis was conducted. It explains the variation of chemical bonding states of the films on different electrode/electrolyte combinations in the device geometry. Fig. 6a–f illustrates the depth-profiling XPS spectra and their deconvolution (Lorentzian–Gaussian) fittings of the Al 2p, O 1s and Ag 3d signals in the PMMA-based devices. Note that, all the XPS spectra were calibrated with reference to the adventitious C 1s peak at 284.5 eV. Fig. 6a and b represents the Al 2p, O 1s signals of the Al/PMMA/Al device after enabling the LRS under forward biasing. The observed Al 2p signals were fitted with two deconvolution peaks of 72.5 eV and 74.7 eV, corresponding to the Al–Al bonding and Al–O bonding of the Al_2_O_3_, respectively.^53^ It is observed that, the area ratio of the Al–O peak to the Al–Al peak increases over sputter-etching times. In particular, the area of Al–O peak increased (marked with up arrow) almost four times higher than the Al–Al peak at 600 s. It demonstrates that the Al atoms from the electrode combine with PMMA at the Al/PMMA interface can give rise to the formation of AlO_*x*_ due to the capture of O atoms from PMMA. Furthermore, the observation of small Al 2p signals at the Al/PMMA interface (at 600 s) can be because of the expansion of the interfacial layer.^54^

The O 1s XPS spectra in Fig. 6b is fitted with three deconvolution peaks located at 530.1 eV (O_i_), 531.2 eV (O_ii_), and 532.3 eV (O_iii_).^54^ The low binding energy component O_i_ is ascribed to the formation of Al–O by the O^2−^ bound to the oxidized Al.^55,56^ The medium binding energy component O_ii_ is attributed to the oxygen vacancies (
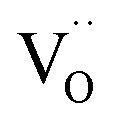
).^57,58^ The high binding energy component O_iii_ is mainly due to the O–H groups of the surface adsorbed H_2_O.^59^ In the initial stage of O 1s spectra, the O_i_ component appears to predominate near the surface of Al electrode and decreases further to the Al/PMMA interface. As the O_i_ peak decreases, the area ratio of the O_ii_ peak is anticipated to increase noticeably. The area of O_iii_ peak related to surface O–H group is decreased with an increase in depth. It is also observed that the O_iii_ and O_ii_ peaks are negatively shifted when the sputter-etching increases, which is mostly due to the electronic interaction between the out-diffused Al atoms and PMMA polymer.^60^ The variation of O 1s bonding states as function of sputter-etching times are in good agreement with the existence of rich AlO_*x*_ layer and 
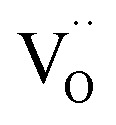
 in the interfacial regime (Fig. 6b). It thus confirms that the formation of 
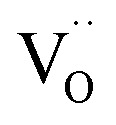
 as conducting channel between the electrodes under LRS.

To further clarify the presence of AlO_*x*_ layer and 
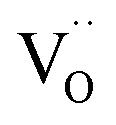
 in the HRS of the device, the depth-profiling XPS scans were also conducted on the Al/PMMA/Al device under pristine state. Fig. S3a and b (ESI†) represents the Al 2p, O 1s signals of the pristine Al/PMMA/Al device. From the Al 2p XPS spectra, it is obvious that intensity of the Al–Al component is substantially higher than Al–O component. Upon the increase in depth, a slight increase in Al–O component is found that may be due to the occurrence of small stoichiometric changes in the Al layer.^54^ Though, the O 1s spectra evidences almost similar peak profiles in the entire depth-profiling studies. The O_i_ peak correspond to the Al–O bonding appeared to be similar from the surface to the interface. The peak area of O_iii_ decreases with an increase in sputter-etching time. It is obvious that the area of the O_ii_ peak is much smaller than both O_i_ and O_iii_ peaks. This is consistent with the insignificant composition of 
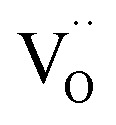
 and AlO_*x*_ layer available in the interfacial region under HRS of the pristine device.^54^

To understand the influence of Ag element on the PMMA device configuration, both AgNP included PMMA and Ag electrode included PMMA devices were also used for depth-profiling XPS analysis. Fig. 6c and d represents the XPS spectra of the Al 2p and Ag 3d signals of the Al/AgNP-PMMA/Al device, respectively. From the Al 2p spectra, it is obvious that the Al–Al component is prominent than the Al–O component in the initial layer. The increase in depth reveals that the increase in area ratio of the Al–O peak to the Al–Al peak. The higher Al–O peak area (marked with up arrow) than the Al–Al peak area is found at 600 s, which indicates the formation of AlO_*x*_ layer in the interface. The corresponding O 1s spectra (Fig. S4 of ESI†) also illustrates the existence of strong O_i_ and O_ii_ components than the O_iii_ component. A slight shift in the peak position is found in the O 1s spectrum while the etch depth increases, that can be due to the interaction between Al and O in the Al/PMMA interfacial region. Essentially, the simultaneous detection of Al 2p and Ag 3d signals at 600 s implies the existence of substoichiometric interfacial layers comprising AlO_*x*_ and Ag in the expanded Al/PMMA interfacial region.^54^ However, the disappearing trend in Al 2p signal and the emerging trend in Ag 3d signal is observed beyond 600 s. The Ag 3d peak arises majorly because of the existence of pre-dissolved AgNP in the PMMA polymer matrix. Fig. 6d shows the Ag 3d XPS spectra of the Al/AgNP-PMMA/Al device. It can be seen that the Ag 3d spectra consists of two major peaks at 368.1 eV and 374.1 eV, corresponding to the Ag 3d_5/2_ and Ag 3d_3/2_ binding energies of metallic Ag (Ag^0^), respectively.^61–63^ As can be seen in Fig. S5 of ESI,† where the Ag 3d doublet peaks are observed after sputter-etching of 600 s, a slight shift in the peak position and change in FWHM is found. According to Busby *et al.*,^64^ this phenomenon can be ascribed to the occurrence of metallic nanoparticle with different particle sizes. The AgNP with smaller diameter mostly exhibit the shift toward higher binding energies. This confirms the formation of Ag conducting metallic bridge between the electrodes with distributed Ag nanoclusters in the interfaces of Al/AgNP-PMMA/Al device.

Fig. 6e and f illustrates the Al 2p and Ag 3d signals of the Ag electrode included PMMA device (Al/Ag/PMMA/Al). It is obvious that the depth-profile exhibits the prominent Al–Al peak with negligibly small Al–O peak. At 600 s, the Al 2p signal is disappeared and Ag 3d signal is emerged, representing the origin of second electrode layer of the device. The binding energies of the Ag 3d_5/2_ doublet peak located at 367.3, eV and 368.1 eV and Ag 3d_3/2_ doublet peak found at 373.3 eV and 374.1 eV after 600 s of depth-profiling studies.^65^ Thereby, it explains the presence of more than one silver species such as metallic Ag and oxidized Ag in the interfaces. It was also estimated that, approximately 94% of metallic silver is present during electrode region. The increase in depth to interfacial regime reduces the metallic silver (∼91%) and enhances the oxidized silver concentration.^65^ This can be due to the interaction between the Ag atom from electrode and O atom from the PMMA polymer. Observation of the decrease in overall intensity of Ag 3d signals from 600 s to 1200 s is mainly because of the variation of electrode region to interfacial region. The corresponding O 1s XPS spectra of the device is shown in Fig. S6 of ESI.† The O_i_, O_ii_ and O_iii_ components almost similar till 600 s of sputter-etching. At 1200 s, a large downshift in the peak positions and change in peak intensity suggests that the change in stoichiometry from electrode to interfacial region.^55^ In particular, the increase in O_ii_ component can be due to the formation of AgO that may create the oxygen deficiencies in the interfaces.^66^

**Fig. 7:**
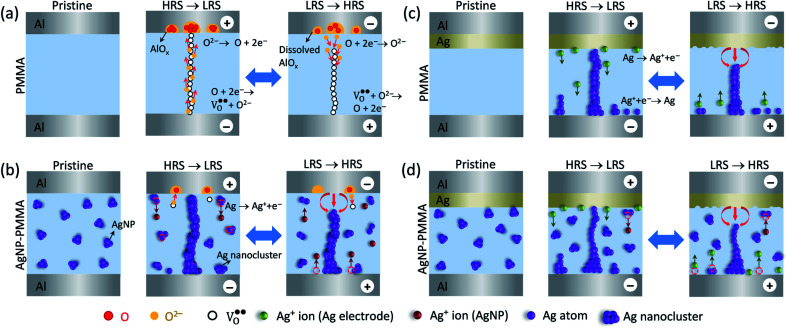
Schematic illustration of the proposed switching mechanisms for (a) Al/PMMA/Al, (b) Al/AgNP-PMMA/Al, (c) Al/Ag/PMMA/Al, and (d) Al/Ag/AgNP-PMMA/Al devices under biasing conditions.

On the basis of the *I–V* characteristics and XPS results, we propose the resistive switching mechanism in the PMMA-based devices, as schematically shown in Fig. 7. In the Al/PMMA/Al device, formation of rich AlO_*x*_ layer in the interfacial region suggests that the resistive switching behavior is associated with the valence change mechanism (VCM).^18,19^ Since VCM cell operates by the formation of oxygen vacancies (
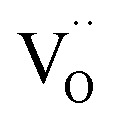
) as conducting bridge between the electrodes under positive biasing, whereas the recombination of oxygen vacancies and oxygen ions (O^2−^) under negative biasing ruptures the conducting bridge (Fig. 7a). The depth-profiling XPS results reveal that the presence of AlO_*x*_ in the active region by the interfacial interaction between Al electrode and PMMA in the Al/PMMA/Al device under biasing. In particular, presence of oxygen atoms in the PMMA can able to react with the out-diffused Al atoms due to the higher oxygen concentration gradient in the interface and higher oxidation tendency of Al.^54,67^ It is thus forms the AlO_*x*_ layer under positive biasing and act as charge trap sites, demonstrating the SCLC mechanism. But, in the case of the Al/AgNP-PMMA/Al device, the resistive switching arises by the electrochemical redox process of the pre-dissolved Ag nanoparticles (AgNP) in the PMMA matrix (Fig. 7b). Under positive bias condition, some of the Ag atoms from AgNP can be oxidized near the positive electrode and migrates Ag^+^ ions into the counter electrode. These Ag^+^ ion migration *via* PMMA can give two possibilities, (1) it can reduce at the counter electrode (Ag^+^ + e^−^ → Ag) and grows the Ag filament towards anode, (2) the reduction process happens at the distributed AgNP in the PMMA interface before reaching to counter electrode, which enlarges the AgNP to Ag nanoclusters.^68,69^ As consequence, the pre-existing AgNP in the PMMA matrix can able to form a metallic bridge between the electrodes with distributed Ag nanoclusters in the interfaces, which enables nonvolatile memory behaviour. Observation of minimum ON state resistance and nonvolatile resistive switching behavior in the Al/AgNP-PMMA/Al device suggests that the resistive switching arises mainly due to the formation of continuous conducting channels. The depth-profiling XPS scans confirm the presence of Ag 3d element in the Al/PMMA interfacial region, which is in agreement with the continuous conducting channel formation between the electrodes. It is also evidenced that, the Al as top electrode used in this device configuration forms a partial AlO_*x*_ layer near the Al/PMMA region, which may influence the trap-controlled current conduction.

Fig. 7c and d illustrates the schematics of the switching mechanism in the Ag electrode included PMMA-based devices. For the Al/Ag/PMMA/Al device, the Ag^+^ ions supplied from the Ag top electrode (TE) alone contribute to the filament formation. Under positive bias condition, this Ag^+^ ions migrate toward the Al bottom electrode (BE), and forms the Ag bridge between the electrodes. Especially, the filament growth takes place at different sites based on the concentration of Ag^+^ ions in the interface.^41^ By taking into account of the *I–V* characteristics of the Al/Ag/PMMA/Al device, large positive voltage always needed to SET the device. It indicates that, less Ag^+^ ion concentration in the PMMA interface causes higher *V*_SET_. Significantly, the inclusion of both Ag electrode and AgNP in the PMMA device (Al/Ag/AgNP-PMMA/Al) enables lower *V*_SET_ as compared to the remaining devices. It indicates that, the Ag^+^ ions injected from both the oxidized Ag TE and pre-dissolved AgNP in the PMMA matrix contribute to the filament growth. Presence of two Ag source in the device configuration can supply large concentration of Ag^+^ ions in the interface, which potentially favours the filament formation and resistive switching characteristics. These results conclude that the combinations of electrode/electrolyte arrangement in the MIM device structure can be able to alter the resistive switching behavior by different conduction mechanism. As a result, the Al/PMMA/Al device exhibits the coexistence of ohmic, Mott–Gurney law and a sudden increase in current regions corresponding to SCLC behavior, which is in contrast to the observation of electric polarization effect and ohmic conduction behavior in the Ag electrode included devices.

## Conclusions

In summary, we demonstrated the resistive switching characteristics and conduction mechanism in the PMMA-based devices with different electrode/electrolyte configurations. From *I–V* studies, the *I*_CC_ range above 50 μA exhibits nonvolatile switching and below this range shows volatile switching behavior in all the four devices. The resistive switching characteristics and conduction mechanism in PMMA-based devices significantly altered when the Al as top electrode is present in the device configuration. The depth-profiling XPS results evidence the formation of AlO_*x*_ in the interfacial region of the Al/PMMA/Al device due to the capture of O atoms from PMMA. Thereby, it confirms the formation of oxygen vacancies as conducting bridge between the electrodes, enabling switching operation. Whereas, the incorporation of Ag element in the PMMA-based device exhibits resistive switching by the metallic Ag filament between the electrodes, interpreting the ohmic behavior in the current conduction. These results contribute toward to understand the importance of device configuration on the resistive switching characteristics of the polymer based MIM devices.

## Experimental

### Materials and methods

#### Fabrication of PMMA-based resistive switching devices

The PMMA-based resistive switching memory devices were fabricated using an ion conducting solid electrolyte sandwiched between two metal electrodes. In our study, both ‘Al’ and ‘Ag’ served as electrode material for the devices. Prior to fabricate the PMMA-based stacked device, SiO_2_ covered (∼300 nm thick) Si substrate (25 × 25 mm^2^) was cleaned using acetone, 2-propanol, and distilled water, separately, under ultra-sonication (∼15 min) process. Also, the substrate was treated in a UV-ozone cleaner for 30 min (home-made system by Santek Electronics, India) prior to coat the bottom electrode (BE). In the Al/PMMA/Al device fabrication, the Al (∼60 nm) as BE was deposited using thermal evaporation technique. Then, the PMMA film was spin coated on the BE. For PMMA film preparation, the materials used as received. Thereby, 100 mg of PMMA (MW = 15 × 10^3^ g mol^−1^, Sigma-Aldrich, India) was dissolved in 7.0 mL of acetonitrile (SRL Chemicals, India). The homogeneous solution was obtained after 45 min of constant stirring at room temperature. Before coating PMMA film on the BE, the film uniformity and thickness was optimized using PMMA to solvent ratio, amount of PMMA solution, and spin coating parameters. Thus, 250 μL of the obtained homogeneous PMMA solution was spin-coated (VB Ceramic Consultants (VBCC), India) on the BE coated substrate at 3000 rpm and 60 s. After spin coating, a film was certainly cured at room temperature. Finally, the Al (∼60 nm) as top electrode (TE) was coated on the PMMA film using thermal evaporation technique. The stacked device exhibits a cross-point structure with an active junction area of approximately 10 × 10 μm^2^.

Similar steps were adopted to fabricate the Al/Ag/PMMA/Al, Al/AgNP-PMMA/Al and Al/Ag/AgNP-PMMA/Al devices. For Al/Ag/PMMA/Al device, the Ag as TE with a thickness of ∼60 nm was coated before coating Al (as protect layer) using thermal evaporation method. In Al/AgNP-PMMA/Al device, the silver nanoparticle (AgNP) with an average particle size of ∼15 nm was used as received (SRL chemicals, India). For the AgNP-PMMA film, 2 wt% of AgNP was additionally incorporated into the PMMA solution, and the remaining procedures were same as mentioned above for film preparation. Owing to the high surface energy of the nanoparticles, there is possibility to agglomerate the AgNP. Hence, the AgNP was ultra-sonicated (∼30 min) before adding into PMMA solution. In the Al/Ag/AgNP-PMMA/Al, the Ag as TE and AgNP incorporated PMMA as electrolyte was used. It is essential to mention that, the constant PMMA film thickness was maintained in all the devices. The polymer film thickness was estimated to be ∼200 nm using atomic force microscopy. Fig. S7 (ESI†) represents the surface topography of the prepared PMMA film.

### Characterization

The current–voltage (*I–V*) measurements were performed using a semiconductor characterization system (Keithley 4200A-SCS) equipped with a home-made probe station setup. The *I–V* measurements were carried out at room temperature and atmospheric conditions. In the entire *I–V* measurements, a constant dual sweep mode was performed for both positive and negative biases. During *I–V* study, the positive bias voltage was applied to the TE and swept with constant sweep rate (5 mV s^−1^), whereas the BE was grounded. In our study, the compliance current (*I*_CC_) was fixed only for the positive bias in order to regulate the ON state current, though the negative bias was swept without fixing certain *I*_CC_ range. The thickness of PMMA film was estimated using AFM (SII E-Sweep/NanoNavi II, in the non-contact mode). The depth-profiling X-ray photoelectron spectroscopy (XPS, ESCALAB 250Xi, Thermo Scientific) was carried out on the PMMA-based MIM devices, which is essential to understand the layer-by-layer chemical bonding states in the electrode and electrolyte regions. For measurement, the depth-profiling scans were done on the device junction area with sputter-etching varied at a rate of ∼0.10 nm s^−1^.

## Conflicts of interest

There are no conflicts to declare.

## Supplementary Material

RA-011-D1RA03561D-s001
