# Supplementary material for: Configurable switching behavior in polymer-based resistive memories by adopting unique electrode/electrolyte arrangement
Source: RSC Adv. 2021 Jul 2;11(38):23400–8. doi: 10.1039/d1ra03561d (PMC9036540; doi:10.1039/d1ra03561d)
Supplement: RA-011-D1RA03561D-s001 [file RA-011-D1RA03561D-s001.pdf]

Electronic Supplementary Material (ESI) for RSC Advances.
This journal is © The Royal Society of Chemistry 2021

Electronic Supplementary Material (ESI) for RSC Advances

Configurable Switching Behavior in the Polymer-based Resistive Memories by Adopting Unique Electrode/Electrolyte Arrangement

[a]Karthik Krishnan*, [a]Shaikh Mohammad Tauquir, [a]Saranyan Vijayaraghavan, and [b]Ramesh Mohan

[a]Corrosion and Material Protection Division, CSIR-Central Electrochemical Research Institute, Karaikudi, Tamil Nadu-630003, India.

[b] Microsystem Packaging Group, CSIR-Central Electronics Engineering Research Institute, Pilani, Rajasthan-333031, India. E-mail: karthikk@cecri.res.in

S. No	Content	Page no.
1	Current–voltage (*I–V*) plot of forming processes	S-2
2	Log *I–V* plot and corresponding linear *I–V* plot	S-3
3	The depth-profiling XPS spectra of Al/PMMA/Al pristine device	S-4
4	O 1s signals of the depth-profiling XPS spectra of Al/AgNP-PMMA/Al	S-4
5	Ag 3d signals of the depth-profiling XPS spectra of Al/AgNP-PMMA/Al	S-5
6	O 1s signal of the depth-profiling XPS spectra of the Al/Ag /PMMA/Al device	S-5
7	Atomic force microscopy (AFM) images of PMMA film	S-6
8	Table: The mean value (μ) and standard deviation (σ) of the V_{SET}/ V_{RESET} and R_{LRS}/R_{HRS}	S-7

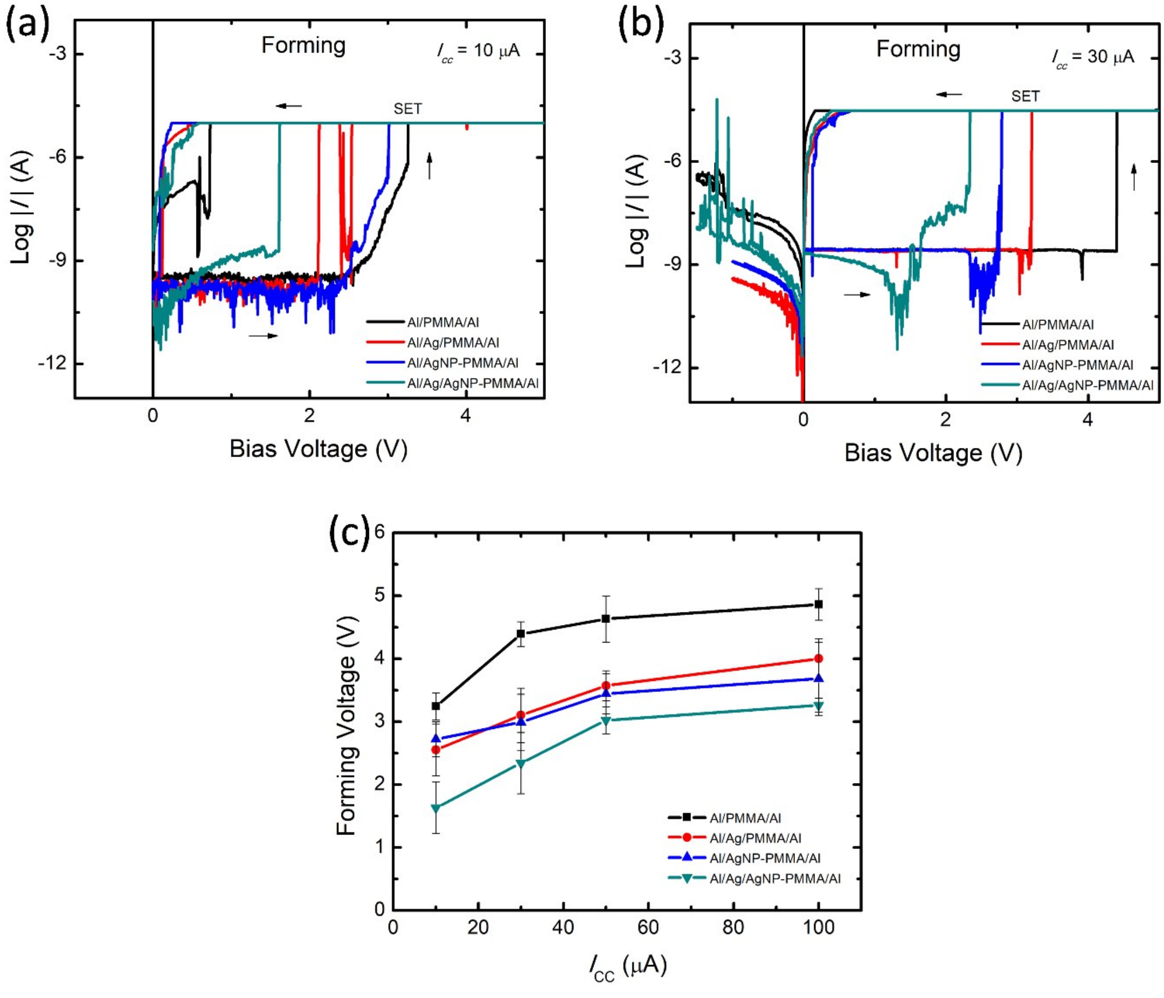

Fig. S1 The *I–V* plots of the forming process of the devices measured under (a) I_{CC} = 10 μA, (b) I_{CC} = 30 μA and (c) the plot between I_{CC} and forming voltage (evaluated from the *I–V* data of 5 devices at each I_{CC}).

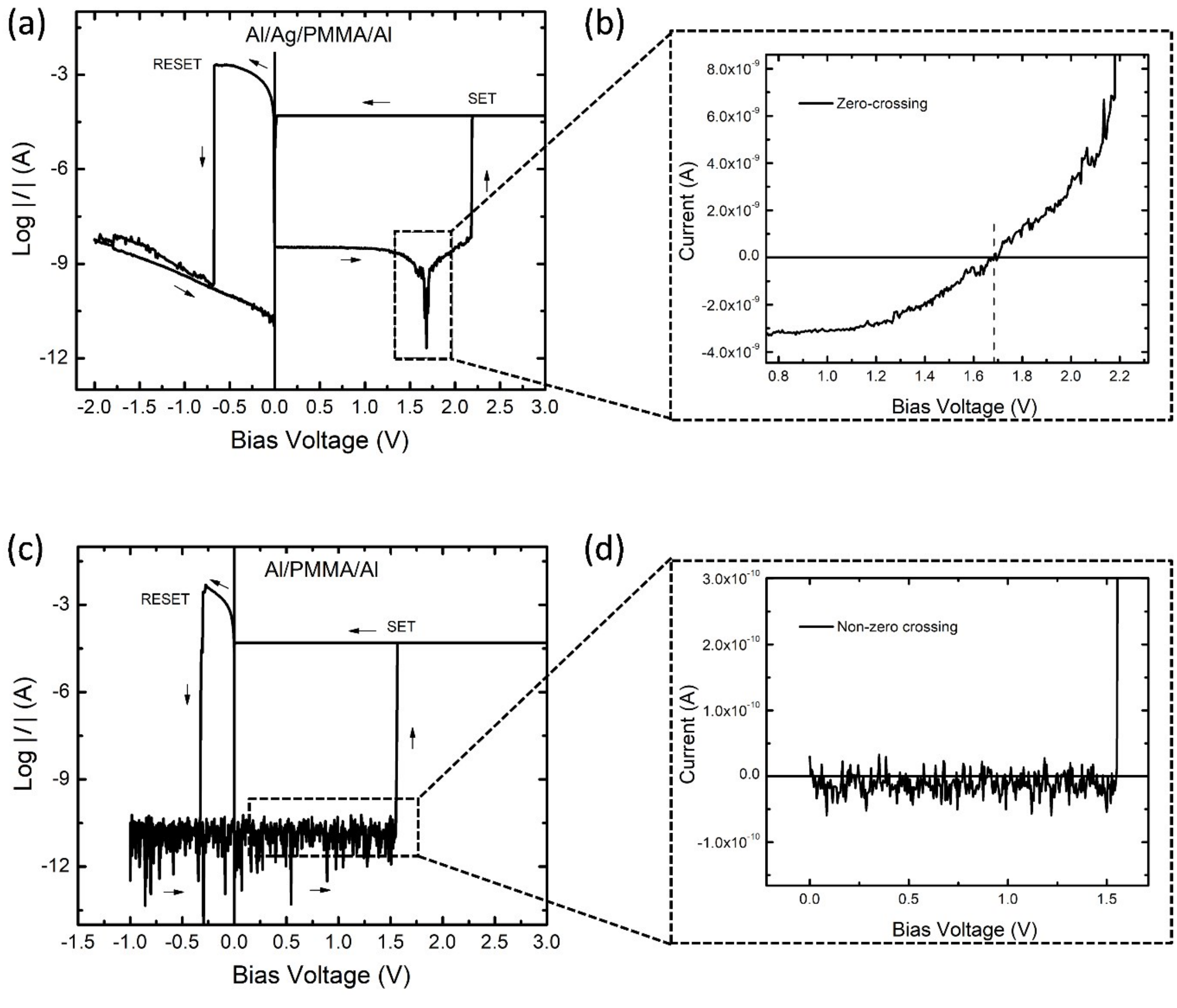

Fig. S2 (a and c) The current minimum in a logarithmic scale of the *I–V* behaviour, corresponding to a (b) zero-current crossing point and (d) non-zero current crossing in a linear scale of the Al/Ag/PMMA/Al and Al/PMMA/Al devices, respectively.

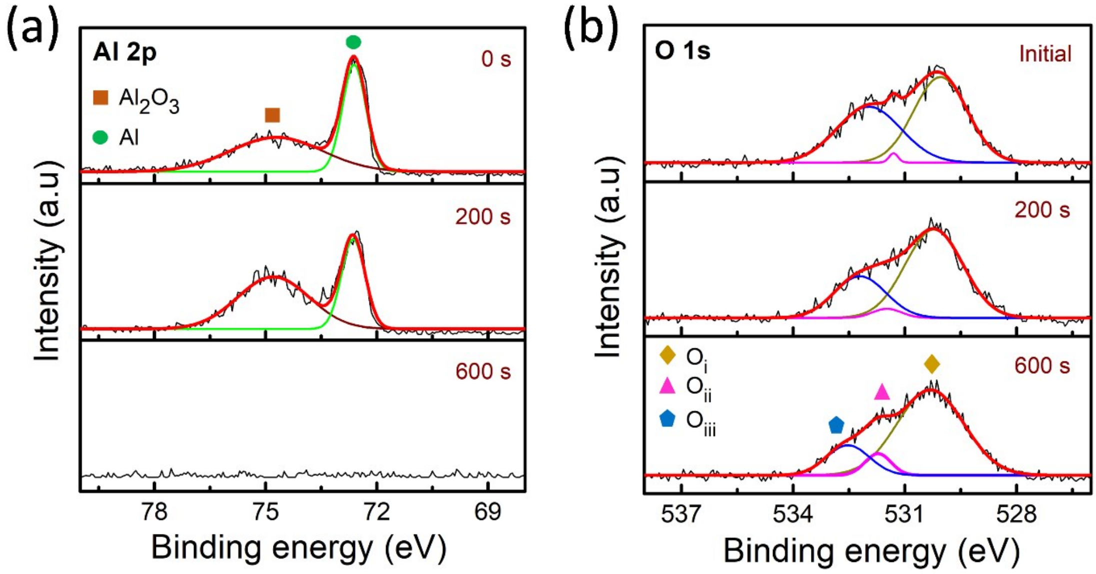

Fig. S3 Depth-profiling XPS scans of (a) Al 2p and (b) O 1s signals of the pristine Al/PMMA/Al device (HRS).

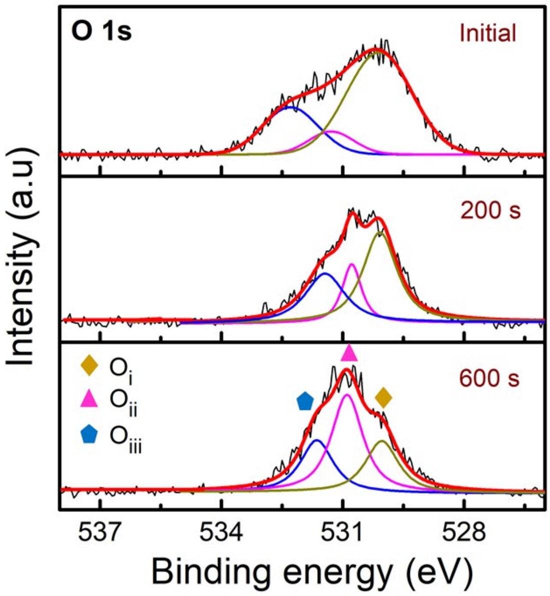

Fig. S4 O 1s signals of the depth-profiling XPS spectra of Al/AgNP-PMMA/Al device.

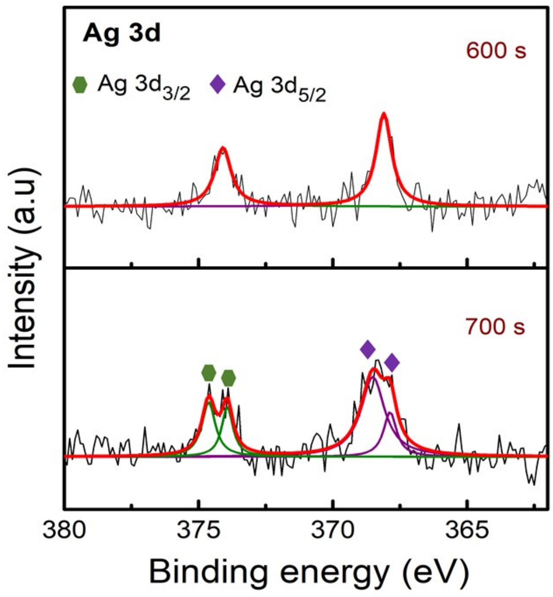

Fig. S5 Ag 3d signals of the depth-profiling XPS spectra of Al/AgNP-PMMA/Al device.

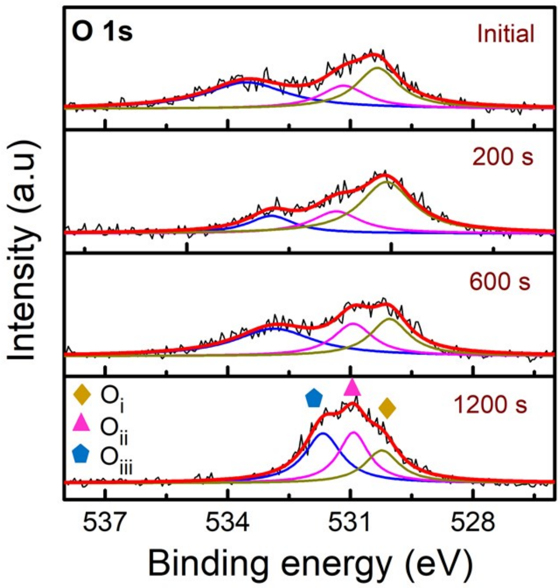

Fig. S6 O 1s signals of the depth-profiling XPS spectra of Al/Ag/PMMA/Al device.

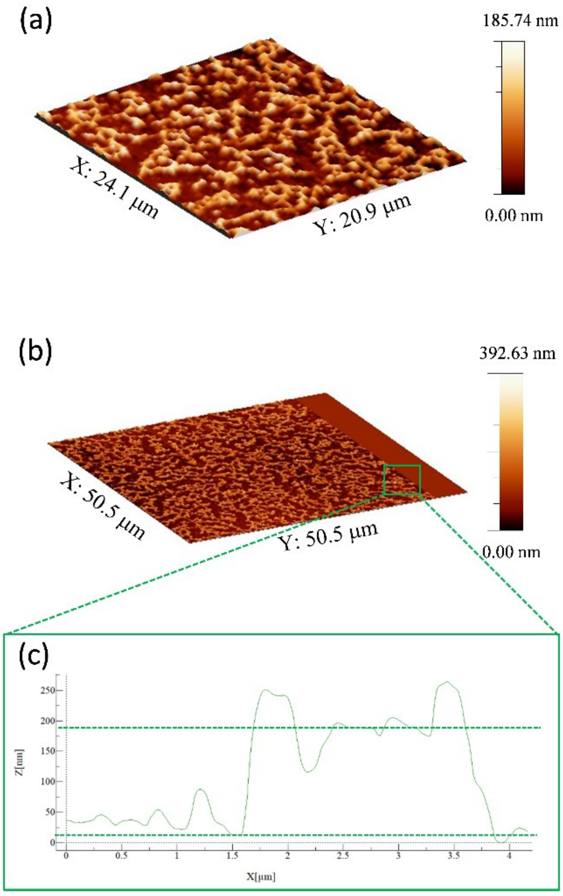

Fig. S7 (a) AFM image of the pure PMMA film and (b) AFM image of the etched film and (c) the corresponding thickness variation profile. The thickness of film is ~200 nm.

Table S1. The mean value (μ) and standard deviation (σ) of the V_{SET}/ V_{RESET} and R_{LRS}/R_{HRS} data of all the devices

**Device**	**Mean value (μ) / Standard deviation (σ)**		**Mean value (μ) / Standard deviation (σ)**	
	V_{SET} (V)	V_{RESET} (V)	R_{LRS} (Ω)	R_{HRS} (Ω)
Al/PMMA/Al	2.28/ 0.70	-0.48/ 0.29	165.7/ 211.6	2.59×10^{9}/ 2.64×10^{9}
Al/Ag/PMMA/Al	2.30/ 0.74	-0.88/ 0.38	218.5/ 210.2	4.58×10^{7}/ 4.94×10^{7}
Al/AgNP-PMMA/Al	2.37/ 0.63	-0.44/ 0.17	67.9/ 117.7	6.50×10^{9}/ 1.16×10^{10}
Al/Ag/AgNP-PMMA/Al	1.73/ 0.61	-0.36/ 0.17	419.8/ 312.1	4.65×10^{7}/ 4.23×10^{7}
